# Effect of sevoflurane and propofol on tourniquet-induced endothelial damage: a pilot randomized controlled trial for knee-ligament surgery

**DOI:** 10.1186/s12871-020-01030-w

**Published:** 2020-05-20

**Authors:** Felipe Maldonado, Diego Morales, Rodrigo Gutiérrez, Maximiliano Barahona, Oscar Cerda, Mónica Cáceres

**Affiliations:** 1 Department of Anesthesia and Perioperative Medicine. Hospital Clínico de la Universidad de Chile. Faculty of Medicine, Universidad de Chile, Santiago, Chile; 2 Program of Cellular and Molecular Biology, Institute of Biomedical Sciences (ICBM), Faculty of Medicine, Universidad de Chile, Santiago, Chile; 3 Centro de Investigación Clínica Avanzada (CICA), Hospital Clínico de la Universidad de Chile. Faculty of Medicine, Universidad de Chile, Santiago, Chile; 4 Department of Orthopaedic Surgery, Faculty of Medicine, Universidad de Chile, Santiago, Chile; 5 Millennium Nucleus of Ion Channels-Associated Diseases (MiNICAD), Santiago, Chile; 6 The Wound Repair, Treatment and Health (WoRTH) Initiative, Facultad de Medicina. Universidad de Chile, Independencia 1027, 8380453 Santiago, Chile

**Keywords:** Syndecan-1, Heparan sulfate, Thrombomodulin, Ischemia reperfusion, Ligament reconstruction

## Abstract

**Background:**

The glycocalyx layer is a key structure in the endothelium. Tourniquet-induced ischemic periods are used during orthopedic surgery, and the reactive oxygen species generated after ischemia-reperfusion may mediate the shedding of the glycocalyx. Here, we describe the effects of tourniquet-induced ischemia-reperfusion and compare the effects of sevoflurane and propofol on the release of endothelial biomarkers after ischemia-reperfusion in knee-ligament surgery.

**Methods:**

This pilot, single-center, blinded, randomized, controlled trial included 16 healthy patients. After spinal anesthesia, hypnosis was achieved with sevoflurane or propofol according to randomization. During the perioperative period, five venous blood samples were collected for quantification of syndecan-1, heparan sulfate, and thrombomodulin from blood serum by using ELISA assays kits. Sample size calculation was performed to detect a 25% change in the mean concentration of syndecan-1 with an alpha of 0.05 and power of 80%.

**Results:**

For our primary outcome, a two-way ANOVA with post-hoc Bonferroni correction analysis showed no differences in syndecan-1 concentrations between the sevoflurane and propofol groups at any time point. In the sevoflurane group, we noted an increase in syndecan-1 concentrations 90 min after tourniquet release in the sevoflurane group from 34.6 ± 24.4 ng/mL to 47.9 ± 29.8 ng/mL (Wilcoxon test, *p* < 0.01) that was not observed in patients randomized to the propofol group. The two-way ANOVA showed no intergroup differences in heparan sulfate and thrombomodulin levels.

**Conclusions:**

Superficial endothelial damage without alterations in the cell layer integrity was observed after tourniquet knee-ligament surgery. There was no elevation in serum endothelial biomarkers in the propofol group patients. Sevoflurane did not show the protective effect observed in in vitro and in vivo studies.

**Trial registration:**

The trial was registered in www.clinicaltrials.gov (ref: NCT03772054, Registered 11 December 2018).

## Background

The glycocalyx layer is a key structure in the regulation of the endothelium. Its dynamic composition results from structural modifications mediated by circulating molecules [1]. A highly sulfated matrix of glycosaminoglycans bound to proteoglycans and glycoproteins acts as a pre-endothelial barrier for macromolecules such as albumin [2, 3], adding a chemical interaction component to the fluid-molecular exchange between blood and the interstitial space [4, 5]. Hence, the interaction with endothelial cells beneath the sub-glycocalyx space creates the endothelial barrier [5].

Loss of the glycocalyx leads to an increase in fluid permeability, interstitial edema, acceleration of leucocyte and platelet adhesion, and coagulation disorders [5, 6]. The endothelial damage is now recognized as the hallmark of different pathologies [2, 7] and is associated with worse outcomes in acute coronary syndrome [8, 9], acute distress respiratory syndrome [10], and sepsis [11].

Perioperative fluid infusions, together with focal and global ischemic episodes, are related to the release of glycocalyx components into the circulation. [4, 12]. Knee-ligament reconstruction surgery with a femoral tourniquet generates a brief ischemic period that enhances the surgical field and prevents major blood loss. A transient ischemia-reperfusion (IR) state is generated after the tourniquet is released. Since reactive oxygen species (ROS) generated after IR mediate the shedding of the endothelial glycocalyx, [13, 14], this surgical scenario predisposes patients to endothelial damage.

Sevoflurane, a general anesthetic, has shown protective endothelial effects both in-vitro and in animal models of IR injury [12, 15–20]. Conversely, propofol does not show this effect and it may further increase the glycocalyx structure damage [4].

We propose that the use of the anesthetic sevoflurane will reduce the IR-induced superficial endothelial damage in comparison with propofol. To test this, we planned a pilot trial to compare the differences in the levels of the superficial endothelial damage biomarker syndecan-1 after tourniquet-induced IR in the presence of general anesthetics. In the same samples, we measured heparan sulfate and thrombomodulin concentrations as additional biomarkers of superficial and profound endothelial damage.

## Methods

### Trial design

We conducted a single-center, randomized, controlled trial with two parallel groups. The trial was approved by the Ethics committee of the Hospital Clínico Universidad de Chile, José Joaquin Aguirre and was conducted according to the principles of the Helsinki Declaration under monitoring by the Good Clinical Practice unit of our institution. Written informed consent was obtained from all patients before they were included in the trial. The outcome assessors were blinded to the group allocation of the patients. This study adhered to CONSORT guidelines for reporting randomized trials [21].

### Participants

We included 16 patients scheduled to undergo elective knee-ligament reconstruction surgery with at least 60 min of tourniquet-induced ischemia of one of the lower extremities at the Hospital Clínico de la Universidad de Chile. The inclusion criteria were age between 18 and 60 years and an American Society of Anesthesiologists (ASA) classification I and II. We excluded patients with allergies to egg or soya, previous history of critical events during surgery and the perioperative period, those at risk of malignant hyperthermia, and patients with 3 or more predictors of difficult airway management.

### Interventions

Blood samples were collected with the informed consent of the patients. All patients entered the operating room and after standard ASA monitorization and placement of an intravenous line (IV), a 50 mL·h^− 1^ Ringer lactate infusion was started. All additional drugs were bolus administered and pushed with 10 mL saline solution. Spinal anesthesia was performed under 1 mg midazolam and 1 μg kg^− 1^ fentanyl sedation. After analgesia and motor block establishment, another mg of midazolam was administered, and according to the study arm allocation, an intravenous or inhalation hypnosis-induction was performed. The airway was secured by laryngeal mask placement before the surgery started. A femoral tourniquet was installed and inflated by the surgeon 120 mmHg above patient systolic blood pressure. Surgery and tourniquet duration, total fluid administration, and the use of vasoactive agents were registered. The sevoflurane group hypnotic anesthetic target was 0.8–1.0 age-corrected minimum alveolar concentration (MAC) and the propofol hypnotic anesthetic target was set to a site-effect target-controlled infusion of 2–2.5 μg·mL^− 1^ (Marsh, keO 1.21 min^− 1^). Both targets allow spontaneous ventilation or pressure support ventilation during surgery. Finally, a femoral nerve block for postoperative analgesia was performed in the post-anesthesia care unit for all patients.

### Blood samples

To measure endothelial damage biomarkers, five different venous blood samples were collected: at the IV placement (baseline value, T1); during surgery before tourniquet release (T2); and 10 (T3), 60 (T4), and 90 (T5) minutes after tourniquet release. At the end of surgery, all patients were transferred to a post-operative care unit where the blood samples were collected. All blood samples were collected by the anesthesiologist team and coded before delivering them to the processing laboratory. Blood samples were incubated for one hour at 37 °C and centrifuged at 1500 rpm for 10 min. Blood serum was stored in a − 80 °C freezer for final analysis.

### Elisa

To characterize the endothelial damage, we decided to measure two superficial biomarkers (syndecan-1 and heparan sulfate) and one deep biomarker (thrombomodulin). To evaluate the biomarker levels in blood serum, we used the following assays: syndecan-1 (CD138) Human ELISA Kit (Catalog #ab46506, Abcam, Cambridge, MA, USA), Heparan Sulfate BioAssay™ ELISA kit (Catalog #356350, USBiological, Salem, MA, USA), and human Thrombomodulin ELISA kit (Catalog #CSB-E07937h, CUSABIO, Houston, TX, USA). All measurements were performed in duplicate. Assays and analyses were conducted according to the manufacturer’s instructions. Standard solutions of syndecan-1, heparan sulfate, and thrombomodulin were provided in each test, with a detection range of 8 ng/mL to 256 ng/mL for syndecan-1, 78 ng/mL to 5 μg/mL for heparan sulfate, and 0.312 ng/mL to 20 ng/mL for thrombomodulin.

### Outcomes

The primary outcome was the difference in syndecan-1 concentrations between sevoflurane and propofol groups 90 min after tourniquet release (T5). Heparan sulfate and thrombomodulin blood serum concentrations were compared between both groups as secondary outcomes. All the comparisons were assessed at the pre-established five points during the perioperative period.

### Sample size

We based our sample size calculation on the normal range values of syndecan-1 reported by Rahbar et al. and on the increase in syndecan-1 levels reported in cardiac surgery patients, septic patients, and trauma injured patients [11, 12, 22]. These findings describe an increase of two or three times to several folds from baseline values. Considering a normal syndecan-1 value of 31.6 ng/mL, a standard deviation of 15.3 ng/mL, and a three-fold increase from the normal range values, a sample size calculation was performed to detect a 25% change in the mean concentration of syndecan-1 with an alpha of 0.05 and a power of 80%. Considering a 20% loss of patients, 16 patients (8 patients per arm) were needed for the two-sided test analysis.

### Randomization

Computer-generated permuted eight-block randomization was performed. With the results, sixteen consecutively sealed and numbered envelopes were generated. Each envelope was opened consecutively after each patient signed the informed consent form. Blood tubes were then labelled with unique patient and sample codes. Sample collectors carried each tube from the operating room to the processing laboratory for analysis.

### Blinding

Sample collectors, laboratory processing investigators, and the outcome assessor were blinded to patients’ allocations. After study termination, the outcome assessor had access to the patient’s sample codes and performed the final analysis.

### Statistical methods

Categorical variables were summarized as relative frequencies. Continuous variables for primary and secondary variables were expressed as the mean and standard deviation (SD) or interquartile rank (IQR). Non-paired results were compared with the Mann-Whitney test. For comparison of repeated assessments, we used a two-way ANOVA. Post-hoc comparisons with baseline values were performed with a Bonferroni correction. For paired data, a Wilcoxon test was performed. Two-tailed *P* values less than 0.05 were considered significant. Data were analyzed with GraphPad Prism software, version 8.0 (La Jolla, CA, USA).

## Results

### Participant flow and recruitment

**Fig. 1:**
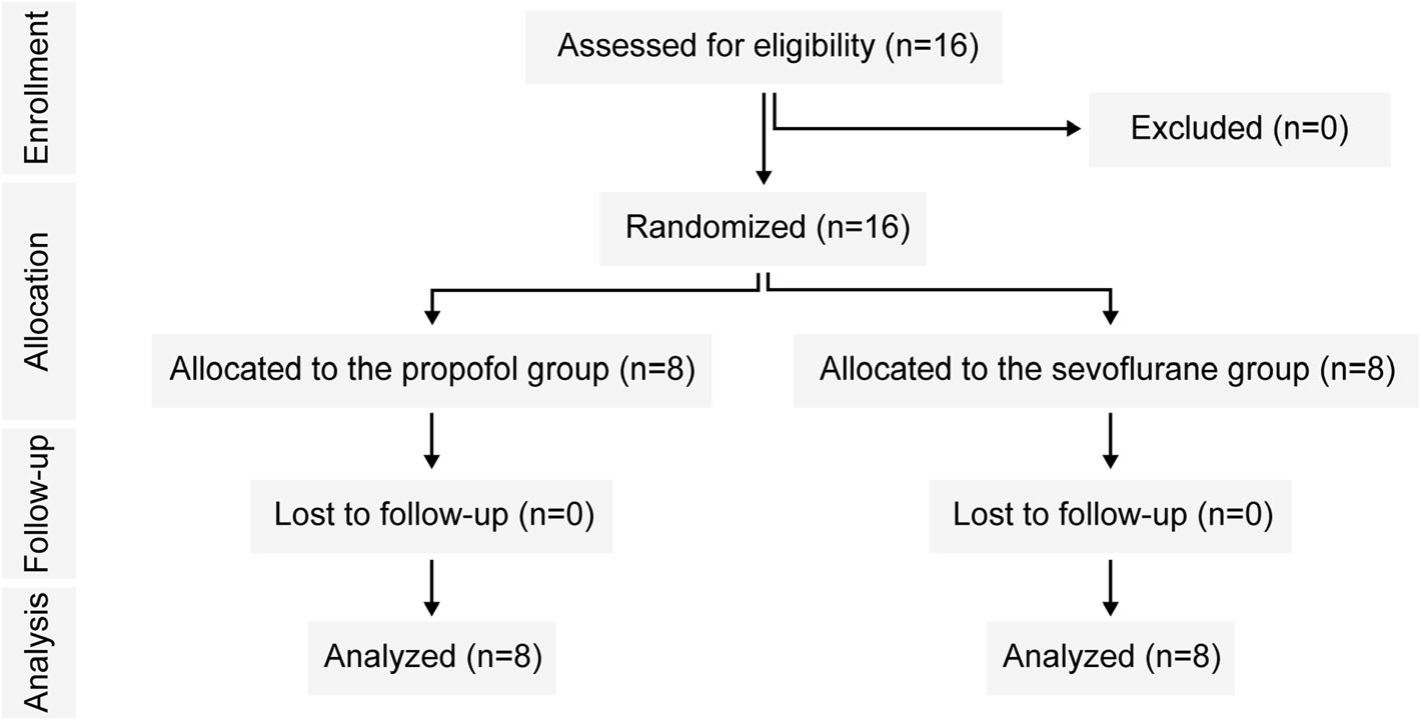
CONSORT diagram

Between December 2018 and July 2019, 16 subjects were consecutively randomized to each of the two arms and analyzed for the primary outcome. There were no exclusions or subject losses in either group (Fig. 1).

### Baseline data

Demographic characteristics are presented in Table 1. There were no differences in age, ASA score, and body mass index. All patients had comparable tourniquet time (96 ± 24 min, Mann-Whitney test, *p* = 0.59) and fluid administration (124 ± 47 mL, Mann Whitney, *p* = 0.08) during surgery. Although there was a higher need for ephedrine in the sevoflurane group, this difference was not significant. One patient required 200 μg of phenylephrine. Blood loss during surgery was described as minimum in all cases. Baseline syndecan-1 concentrations were 34.61 ± 24.4 ng/mL in the sevoflurane group and 27.02 ± 12.61 ng/mL in the propofol group.

**Table 1:** Patient characteristics. Baseline and surgical characteristics of patients randomized to the sevoflurane and propofol groups. BMI (body mass index), ASA (American Society Association), n (number of subjects), Standard deviation (SD)

	Total (SD) (***n*** = 16)	Propofol (SD) (***n*** = 8)	Sevoflurane (SD) (n = 8)
**Age**	27 (9)	25 (6)	30 (11)
**BMI**	28 (4)	27 (4)	29 (5)
**ASA Score (n)**			
**1**	11	5	6
**2**	5	3	2
**Tourniquet Time (min)**	96 (24)	91 (17)	101 (30)
**Fluid dose (mL)**	124 (47)	105 (33)	144 (52)
**Number of patients with need of Ephedrine**	8	3	5
**Ephedrine dose (mg)**	23 (17)	14 (9)	28 (19)
**Number of patients with need of Phenylephrine**	1	0	1
**Phenylephrine dose (mg)**	200	0	200

### Outcomes

#### Main outcome (syndecan-1)

**Fig. 2:**
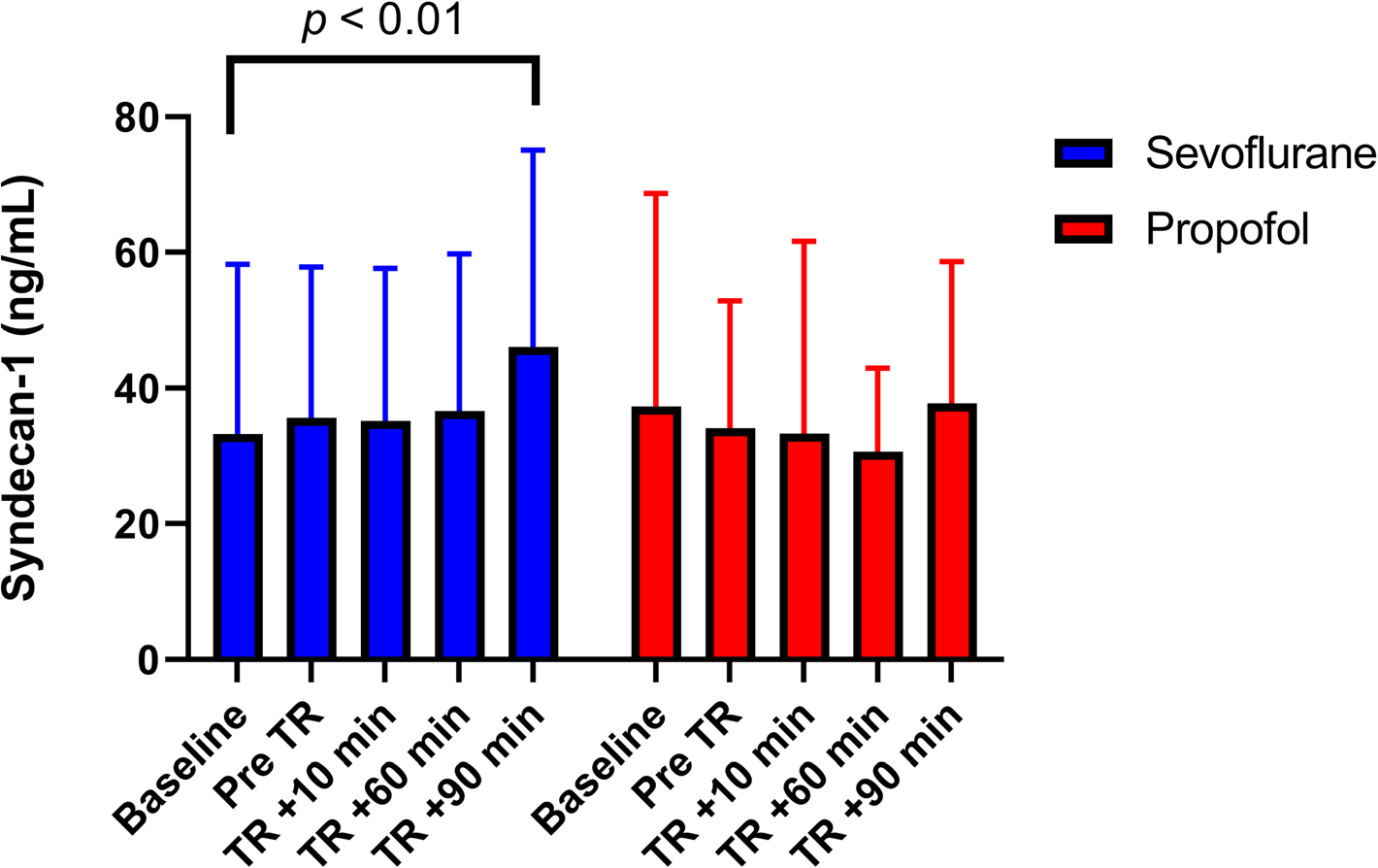
Endothelial damage biomarkers differences between sevoflurane and propofol groups. Endothelial damage was observed as an increase in the syndecan-1 concentration in the sevoflurane group. The difference was significant from baseline values at 90 min after tourniquet release **(**Wilcoxon test, p < 0.01). There was no difference between sevoflurane and propofol groups

Our primary outcome was the difference in the syndecan-1 concentrations between sevoflurane and propofol groups. In a two-way ANOVA with Bonferroni correction, we found no differences in the plasma levels of syndecan-1 between the sevoflurane and propofol groups at any time. However, when we analyzed the changes in syndecan-1 levels across different times in each group, we found that the syndecan-1 level was elevated 90 min after tourniquet release in comparison to the baseline values in the sevoflurane group (from 34.6 ± 24.4 ng/mL to 47.9 ± 29.8 ng/mL, Wilcoxon test, *p* < 0.01). This increase was not found in the propofol group (Fig. 2).

#### Secondary outcomes (heparan sulfate and thrombomodulin)

**Fig. 3:**
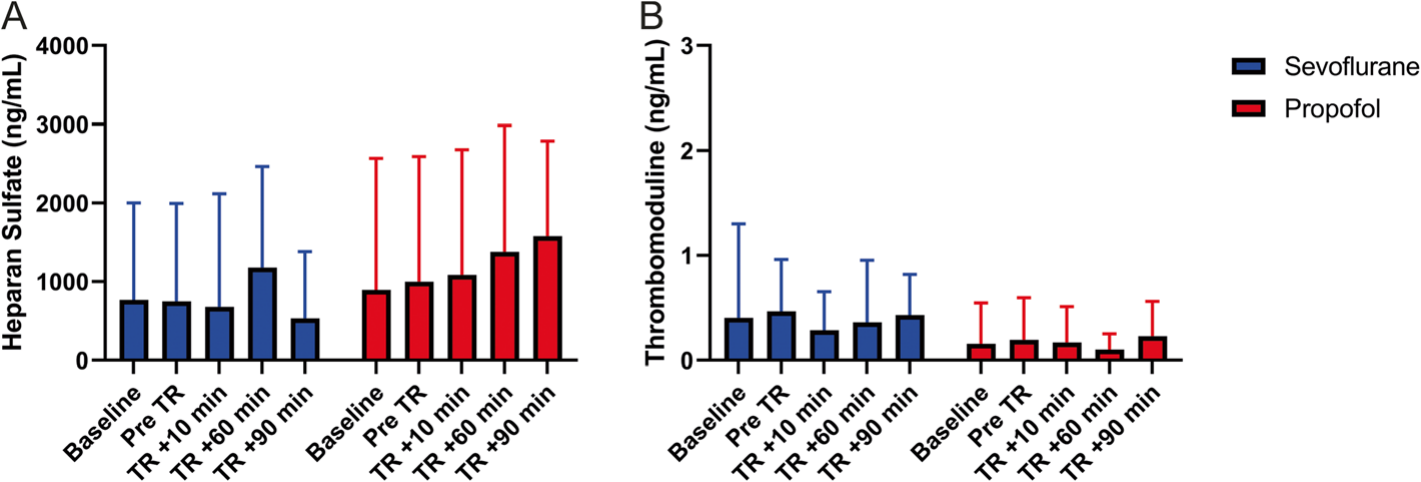
Heparan sulfate and thrombomodulin differences between sevoflurane and propofol groups. Endothelial damage measured by heparan sulfate (**a**) did not show differences in the serum concentration at any time point. The endothelial cell integrity was similar in both groups as thrombomodulin did not show serum elevation (**b**)

Heparan sulfate and thrombomodulin did not differ significantly between groups at the time points evaluated (Fig. 3).

### Other analysis

Our power analysis allowed us to identify differences in the syndecan-1 concentration, and we performed a pooled examination of our cohort’s data to describe the behavior of perioperative serum biomarkers in this surgery. We observed a baseline syndecan-1 concentration of 30.9 ± 19.6 ng/mL and a non-significant increase in the concentration during surgery over the next 60 min following tourniquet release. After 90 min (T5), a 37% increase in syndecan-1 concentration was observed (42.7 ± 25.5 ng/mL; Wilcoxon test, *p* < 0.001). This increase was also significantly different from all the other sampling periods (one-way ANOVA, T5 vs. T2, *p* = 0.013; T5 vs. T3, *p* = 0.019, T5 vs. T4, *p* = 0.013). Baseline heparan sulfate concentration was 919.4 ± 1579.8 ng/mL. Like syndecan-1, heparan sulfate concentration increased after tourniquet release, and after 60 min a 58% increase was reached (1588.7 ± 1692.1 ng/mL, Wilcoxon test, *p* = 0.036). This elevated concentration remained at 90 min after tourniquet release, but there were no significant differences from baselines values. In contrast to the elevations of both superficial endothelial biomarkers, thrombomodulin levels did not show any elevation during the perioperative period.

**Fig. 4:**
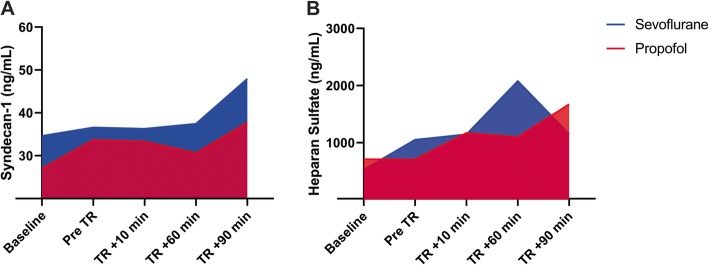
Area under curve for syndecan-1 and heparan sulfate during the perioperative period. Representation of the area under the curve analysis. Amounts of Syndecan-1 (**a**) and Heparan sulfate (**b**) released in the sevoflurane and propofol groups during the study protocol. Anesthetics showed no differences in the levels of biomarker released during the study

Finally, to estimate whether the anesthetic choice modifies the amount of each biomarker released during the study protocol, an area under the curve analysis was performed. Sevoflurane and propofol showed no differences in the levels of biomarker released during the study (syndecan-1, sevoflurane: 5836 ng [IQR: 3264–8829 ng], propofol: 5596 ng [IQR: 2319–7202], Mann-Whitney test, *p* = 0.295; heparan sulfate, sevoflurane: 224407 ng [IQR: 147003–315,892 ng], propofol: 127677 ng [IQR: 36996–291,879], Mann-Whitney test, *p* = 0.257; thrombomodulin, sevoflurane: 34 ng [IQR: 10–87 ng], propofol: 4.8 ng [IQR: 0–34], Mann-Whitney test, *p* = 0.058) (Fig. 4). A significant difference was observed in the amount of thrombomodulin released in the propofol group after correcting the results by ischemic-tourniquet time (thrombomodulin, sevoflurane: 17 ng [IQR: 2–63 ng], propofol: 0.2 ng [IQR: 0–9], Mann-Whitney test, *p* = 0.020).

### Adverse effects

There were no adverse or unintended effects observed in the subjects.

## Discussion

In the present article, we used a serum biomarker measurement approach to evaluate the protective effect of two common anesthetics used in clinical practice on the endothelial glycocalyx-cell integrity after a tourniquet-induced ischemia-reperfusion period. The presence of circulating syndecan-1 and heparan sulfate, both components of the glycocalyx, was used as a marker of early superficial endothelial damage. Thrombomodulin, a profound endothelial damage marker, was used for quantification of cell layer damage in the endothelium [8, 10].

We observed an increase in superficial-endothelial damage biomarkers after tourniquet release in subjects randomized to sevoflurane that were not observed in the propofol group. The pooled analysis shows that both syndecan-1 and heparan sulfate levels were elevated after tourniquet release in knee ligament surgery. Interestingly, although thrombomodulin biomarker concentrations did not increase, the AUC analysis showed a significant difference in the amount of the biomarker released between groups. This may imply that endothelial cell damage may occur and may be affected by anesthetics. More studies will be needed to confirm this observation.

The reported normal values for plasma syndecan-1 are in the range of 0 to 300 ng/mL, while those for heparan sulfate range from 4820 to 7940 ng/mL. The study by Rehm et al. found a basal plasma syndecan value of 12 ng/mL and 5900 μg/mL for heparan sulfate [12]. The study by Johansen et al. in septic patients describes a normal range of serum syndecan-1 of 51 ± 12 ng/mL and a range of 4.5 ± 0.8 ng/mL for thrombomodulin[11]. The study by Rahbar et al. reported that the reference syndecan-1 value in healthy volunteers provided by Abcam was 31.6 ± 15.3 ng/mL [22]. Our results showed baseline syndecan-1 concentrations of 34.61 ± 24.4 ng/mL in the sevoflurane group and 27.02 ± 12.61 ng/mL in the propofol group, both of which were within the range of previous reports [11, 22]. In the study by Rehm et al., cardiopulmonary bypass during ascending aorta surgery, infrarenal aortic aneurysm, and circulatory arrest resulted in a several-fold increase in the levels of endothelial damage biomarkers. In our work, we found that the transient ischemic period of a lower extremity induced a 37% increase in syndecan-1 levels. This may be explained by the substantially less ischemic tissue than in the cardiac surgery scenario.

In vitro and in vivo studies have suggested that sevoflurane anesthesia has a glycocalyx protective effect [4, 13, 15–18, 23, 24]. Recently, Kim et al. compared the effects of sevoflurane and propofol in 78 patients who underwent thoracic surgery [25]. They found an increase in the heparan sulfate and syndecan-1 plasma concentrations during one-lung ventilation surgery. Although there was an increase in both biomarkers, there was no significant difference in anesthetic groups. These findings are consistent with our study since we found no differences between the sevoflurane and propofol groups. Nevertheless, we observed that syndecan-1 levels were significantly higher in the sevoflurane group in comparison with the baseline values and, this was not observed in the propofol group. Moreover, the sevoflurane-associated increases in syndecan-1 levels accounted for the main component of the increment observed in the pooled data.

Thrombomodulin is a cell transmembrane glycoprotein marker of endothelial disruption. Its liberation into plasma is a predictor of multiorgan failure and death [11]. Since thrombomodulin elevation reflects profound endothelial damage, we were able to separate the endothelial layer impact of the ischemia-reperfusion insult and verify that it mainly affected the glycocalyx layer.

There are a few interventions that may reduce glycocalyx-endothelial damage. Limiting the fluid administration and use of methylprednisolone has been shown to reduce the elevation of syndecan-1 levels [26–28]. We limited our fluid infusion to an arbitrary low dose of 50 mL·h^− 1^ in both groups and we avoided the use of any corticosteroid as an antiemetic during the perioperative blood sampling period. With this strategy, we did not observe a significant elevation in any biomarker level before the tourniquet release during surgery. This approach may be suitable for all surgeries where no fluid loss is expected.

Many insults that increase the endothelial damage are related to a surgical scenario. Procedures such as cardiac surgery with extracorporeal circulation [29], fluid administration [30, 31], and one-lung ventilation [19] are performed under general anesthesia, so the studies identifying the best pharmacologic approaches are crucial. Various anesthetics have been studied for their potential protective effect. In vitro and in vivo models have demonstrated the protective effect of sevoflurane against ROS on the endothelium [13, 15, 17, 18, 23]. Propofol did not show a protective effect; moreover, one study on isolated guinea pig hearts reported an increase in syndecan-1 levels after 20 min of ischemia [4]. These findings have been difficult to translate to clinical practice since the protective effect of sevoflurane observed in different organ studies has not been observed in patients [25].

We use a novel model for studying tourniquet-induced endothelial damage. We found no previous studies in the knee-traumatological scenario. Moreover, knee-ligament surgery has not been described before as a procedure leading to endothelial damage. Most articles report that a collapsed lung during one-lung ventilation surgery generates an elevated inflammatory response with increased production of ROS [19, 20, 23]. Here, we describe that the ischemic insult after tourniquet release is enough to induce a biomarker signal of injury. Our cohort was composed of healthy ASA I and II subjects in which this surgery involves little morbidity per se. Since syndecan-1 elevation observed in septic and critical care patients are associated with higher morbidity and mortality, is relevant to find an adequate strategy to avoid endothelial destruction as it is implicated in tissue edema, coagulopathy, and organ dysfunction as a great number of critical patients would need surgery. Anesthetic selection may be crucial in their treatment if there are insults that generate ROS and endothelial damage.

### Limitations

Here, we compare the effects of two common anesthetics on endothelial damage. We analyzed five different points, covering the perioperative period. We found that the increase in Syndecan-1 levels at 90 min post-tourniquet release was the clearest moment to detect differences with baseline values. This work has several limitations. First, we included a small population sample (16 patients) that allowed us to detect a 25% change in the mean concentration of syndecan-1, and a broader sample may be necessary to detect smaller differences and confirm our findings. Another limitation is that we decided to compare general anesthetics in addition to a spinal technique. In this scenario, we do not know how spinal anesthesia and the related vasodilation may impact the reperfusion phenomenon or if this shows a markedly significant difference between the two groups. We used only one type of surgery assuming that the ischemic insult of one extremity may be enough to elicit post-ischemic-reperfusion ROS generation and systemic endothelial damage. Since this is a rapid phenomenon, we decided to measure biomarker concentrations in the early reperfusion periods until 90 min post tourniquet release. Although we observed an increase in biomarker levels at 90 min after tourniquet release, studies of biomarker concentrations further in time may have helped us understand if syndecan-1 levels show more differences between groups, and this may have strengthened our results. Finally, a measure of ROS may be crucial to establish a correlation between both reperfusion and biomarker release.

## Conclusions

Contrary to our hypothesis, patients in the sevoflurane group showed an increase in the serum concentration of syndecan-1. Our results indicate that the use of propofol as a hypnotic may be a reasonable choice during knee-ligament surgery and in association with limited fluid administration may have minimal effect on the endothelium.

Finally, we observed that after periods of ischemia-reperfusion in lower extremities induced by a tourniquet, there was an increase in the serum concentrations of syndecan-1 and heparan sulfate, but not thrombomodulin. This may reflect superficial endothelial damage without alterations in the cell layer integrity. Sevoflurane did not show the protective effect observed in in vitro and in vivo studies.

## Data Availability

The datasets used and/or analyzed during the current study are available from the corresponding author on reasonable request.

## Abbreviations

ASA: American Society Association
IQR: Interquartile rank
IR: Ischemia-reperfusion
IV: Intravenous line
MAC: Minimum Alveolar Concentration
ROS: Reactive oxygen species
SD: Standard deviation
